# Diabetes does not affect motor recovery after intracerebral hemorrhage

**DOI:** 10.1515/tnsci-2020-0125

**Published:** 2020-08-25

**Authors:** Seung Hwa Jang, Sang Gyu Kwak, Min Cheol Chang

**Affiliations:** Department of Rehabilitation Medicine, College of Medicine, Yeungnam University, 317-1, Daemyungdong, Namku, 705-717, Daegu, Republic of Korea; Department of Medical Statistics, College of Medicine, Catholic University of Daegu, Daegu, Republic of Korea

**Keywords:** diabetes, motor outcome, diffusion tensor tractography, intracerebral hemorrhage

## Abstract

**Background:**

This retrospective study evaluated whether diabetes affects motor outcome after stroke by analyzing the effects of diabetes on motor prognosis by controlling for critical factors, including lesion type and location, corticospinal tract (CST) state, patient age, lesion volume, and treatment method during the stroke.

**Methodology:**

We recruited 221 patients with intracerebral hemorrhage (ICH) of the basal ganglia. We used diffusion tensor tractography to investigate the CST state. We also evaluated the hemorrhage volume. We obtained information on the presence of diabetes and age by chart review. Motor outcomes at 6 months were measured using the upper and lower limb motricity index (MI), modified Brunnstrom classification (MBC), and functional ambulation category (FAC). We used multiple linear regression tests to investigate whether diabetes affected motor outcomes after stroke after adjusting for other factors, including CST state, age, lesion volume, and treatment method.

**Results:**

The presence of diabetes was not correlated with motor outcome measurements, including upper and lower MIs, MBC, and FAC, at 6 months after the onset. However, the CST state, age, lesion volume, and treatment method were significantly correlated with nearly all motor outcomes.

**Conclusions:**

We found that diabetes did not significantly affect motor outcomes after ICH.

## 1 Introduction

Motor deficits are a major sequela after stroke [1,2,3], hindering patient’s independent activities of daily living and deteriorating the quality of life [3]. After stroke, more than half of the patients have significant motor deficits. Several factors including the state of motor-related neural tracts, lesion location, patient age, and treatment method reportedly affect motor outcomes after stroke [2,4,5,6,7]. While previous studies have evaluated the effects of diabetes on motor recovery after stroke [8,9,10], this topic remains controversial. Most of these studies did not control for factors that can affect the motor recovery of stroke patients [7,8,9,10], which may have contributed to the unreliable conclusions.

The current study aimed to accurately evaluate the effects of diabetes on motor recovery after stroke by recruiting only patients with intracerebral hemorrhage (ICH) in the basal ganglia (BG). Furthermore, we controlled for critical factors, including the state of the corticospinal tract (CST), age, lesion volume, and treatment method. We also evaluated the CST state using recently developed diffusion tensor tractography (DTT) derived from diffusion tensor imaging (DTI).

## 2 Materials and methods

### 2.1 Subjects

This retrospective study recruited 221 patients admitted for rehabilitation at the Department of Rehabilitation in a university hospital according to the following criteria (Table 1): (1) first-ever stroke, (2) age 20–80 years, (3) severe weakness of the affected extremities to the degree of being unable to move the affected limb without gravity and complete weakness of the affected hand (finger flexor and extensor) and ankle (ankle dorsiflexor and plantar flexor) within 24 h of onset, (4) a hematoma involving the BG seen on brain computed tomography (CT) with confirmation by a neuroradiologist (with the hematoma epicenter within the BG), (5) DTI scanning > 7 days after onset, and (6) the absence of serious medical complications such as cardiac problems or pneumonia, from onset to final evaluation. Patients with apraxia or severe cognitive problems were excluded.

**Table 1:** Demographic data and 6 month motor outcomes of the included patients

Age, years	53.8 ± 12.2
Sex (M:F), *n*	133:88
Lesion side (R:L), *n*	117:104
Hemorrhage volume, mL	29.9 ± 23.2
Surgery:conservative treatment, *n*	111:110
Diabetes:nondiabetes, *n*	44:177
From onset to DTT evaluation, day	47.6 ± 80.7
Preserved CST:interrupted CST	91:130
Initial MBC	1.0 ± 0.0
Initial FAC	0.0 ± 0.0
UMI (6 months after onset)	52.4 ± 26.4
LMI (6 months after onset)	59.4 ± 23.5
MBC (6 months after onset)	3.6 ± 2.0
FAC (6 months after onset)	3.0 ± 1.5

Abbreviations: DTT, diffusion tensor tractography; CST, corticospinal tract; MBC, modified Brunnstrom classification; FAC, functional ambulation category; MI, motricity Index.

Values are presented as mean ± standard deviation.


**Informed consent:** Informed consent has been obtained from all individuals included in this study.
**Ethical approval:** The study protocol was approved by the Institutional Research Board of the university hospital. The research related to human use has been complied with all the relevant national regulations, institutional policies, and in accordance with the tenets of the Helsinki Declaration.

### 2.2 Clinical evaluation

Motor function was measured twice in each patient: at ICH onset and at 6 months after ICH onset. Motricity indices (MIs) were used to measure motor function (maximum score: 100) [11]. The functions of the affected hands were categorized using the modified Brunnstrom classification (MBC) (score range: 1–6) [12,13]. Walking ability was quantified using the standardized functional ambulation category (FAC) score (range: 0–5), which is based on characterizations of the levels of assistance required during a 15 m walk [14]. Higher MI, MBC, and FAC scores indicated better motor function.

### 2.3 DTI acquisition and CT analysis

DTI was performed using a sensitivity-encoding head coil on a 1.5-T Philips Gyroscan Intera (Hoffman-LaRoche, Ltd, Best, the Netherlands) with single-shot echo-planar imaging and navigator echo. Sixty contiguous slices (matrix = 128 × 128, field of view = 221 × 221 mm^2^, TE = 76 mm, TR = 10,726 ms, SENSE factor = 2; EPI factor = 67 and *b* = 1,000 s/mm/mm, number of excitations = 1, with a 2.3 mm slice thickness) were acquired for each of the 32 noncollinear diffusion-sensitizing gradients. The scanning time for DTI acquisition was 7 min 32 s. Head motion effects and image distortions due to eddy currents were corrected by multiscale affine two-dimensional (2D) registration.

**Figure 1:**
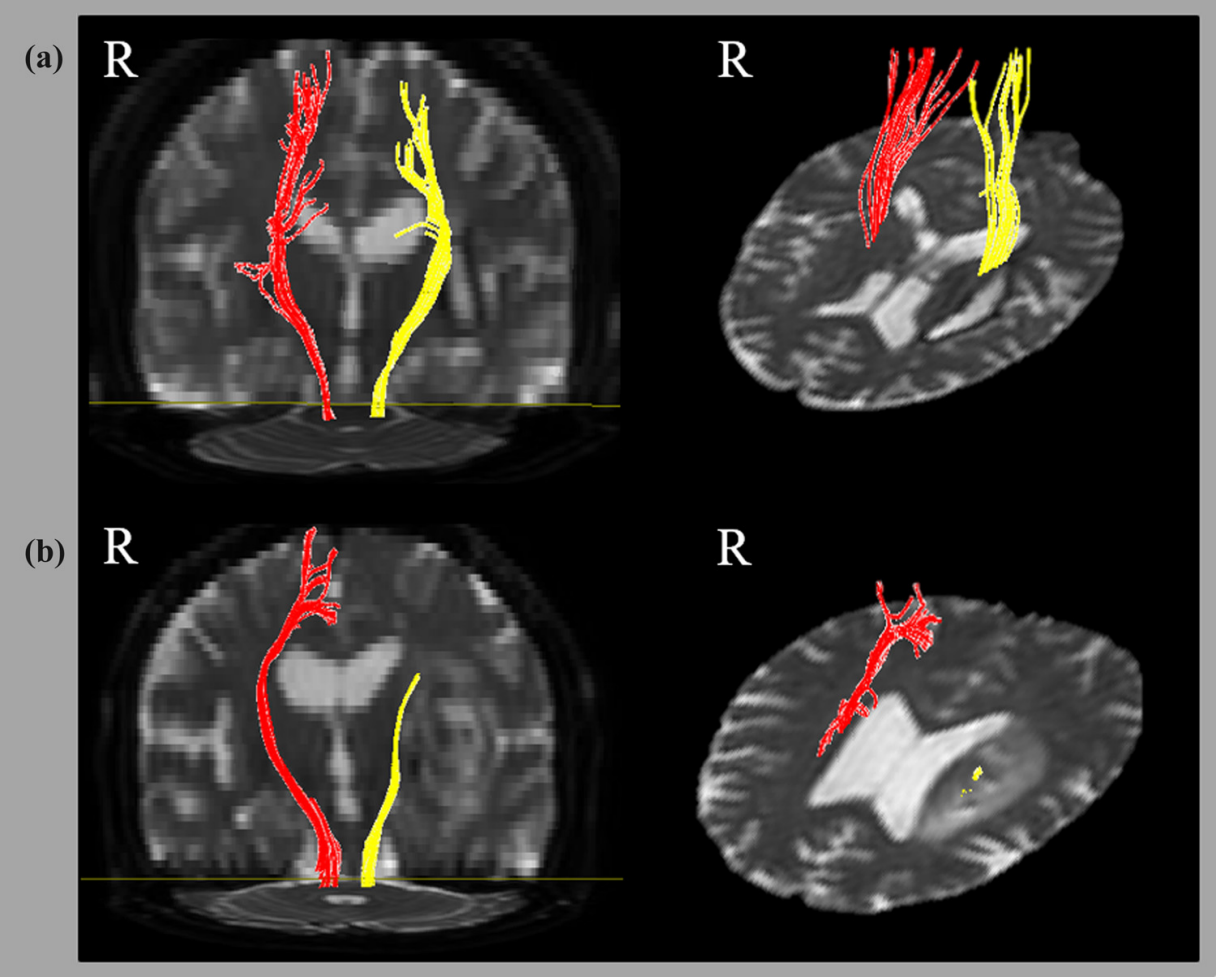
DTT findings of the CST. (a) A 51 year-old woman with CST preservation around the hematoma on the BG. The CST originated from the cortex of the affected hemisphere and passed around the hematoma to the medulla. (b) A 52 year-old woman with interrupted CST at the hematoma on the BG. The tract does not reach the cortex of the affected hemisphere.

Fiber connectivity was evaluated using fiber assignment by continuous tracking, a three-dimensional fiber reconstruction algorithm within the Philips PRIDE software (Philips Medical Systems, Best, the Netherlands). CST fiber tracking was performed using a fractional anisotropy (FA) threshold of >0.2 and a direction threshold of 60° [7]. In each case, a seed region of interest (ROI) was drawn in the CST portion of the anterior mid-pons on a two-dimensional (2D) FA color map; another ROI was also drawn in the CST portion of the anterior low-pons on a 2D FA color map. Fiber tracts passing through both ROIs were designated as the final tracts of interest (Figure 1). According to the CST integrity, the DTT findings were divided into either CST + (91 patients), in which the CST was preserved around the BG hematoma, that is, the CST originated from the cortex of the affected hemisphere and passed around the hematoma to the medulla, or to CST− (130 patients), in which the CST was interrupted at, or around, the hematoma.

### 2.4 Evaluation of the presence of diabetes

The patients were divided into two groups according to the presence of diabetes: DM+ (44 patients) and DM− (177 patients). The criteria for classifying patients into the DM+ group were fasting plasma glucose level ≥126 mg/dL, plasma glucose at 2 h after 75 g oral glucose load ≥200 mg/dL, HbA1c ≥ 6.5%, random plasma glucose ≥200 mg/dL with classic hyperglycemic symptoms, and previous diagnosis of type 2 diabetes [15]. Patients with type 1 diabetes were excluded from this study.

### 2.5 Estimation of hematoma volume

Hematoma volumes were calculated using initial CT scans. The largest diameters of the hematomas (*A*), the largest diameters of the hematoma perpendicular to *A* (*B*), and heights of hemorrhages were measured in centimeters (*C*). The formula used to estimate hematoma volume (mL) was as follows: hematoma volume = 4π/3 × (*A*/2) × (*B*/2) × (*C*/2) [16].

### 2.6 Statistical analysis

Data were analyzed using the IBM SPSS Statistics for Windows, version 23.0 (IBM Corp., Armonk, NY, USA). We performed multiple linear regression analysis to investigate whether diabetes affected motor outcomes after stroke. We presented the values of the regression coefficient, *t*-value, and *p*-value for each independent variable and determined the coefficient, *F*-value, and *p*-value for each regression model. The independent variables were the presence of diabetes, CST state, lesion volume, age, and treatment administered during ICH emergence (surgery or conservative treatment). The categorical variables (presence of diabetes and CST state) were converted into binary variables. For diabetes, the values were 1 and 0 if diabetes was present and absent, respectively. Regarding CST, the value was 1 if the CST was preserved and 0 if it was interrupted. Among treatment methods, the value was 1 if a surgery was performed and 0 if conservative treatment was used. In addition, we evaluated the correlation between the glycated hemoglobin (HbA1c) level and motor outcomes at 6 months after ICH onset using Pearson’s correlation tests. Statistical significance was defined as *p* values <0.05.

## 3 Results

The demographic data of the included patients are listed in Table 1 and Table S1. The average HbA1c value in patients with diabetes was 7.4% ± 1.6. The results of multiple linear regression analyses are listed in Table 2. The presence of diabetes was not correlated with motor outcome measurements, including upper MI (UMI) and lower MI (LMI), MBC, and FAC, at 6 months after onset (*p* > 0.05). However, the CST state, age, and lesion volume were significantly correlated with all motor outcomes (*p* < 0.05). The applied treatment method was correlated with UMI and LMIs and MBC (*p* < 0.05). CST preservation, younger age, smaller lesion volume, and surgery were predictive factors for good motor prognosis.

**Table 2:** Results of multiple regression analysis for UMI, LMI, MBC, and FAC

Dependent variable	UMI			LMI		
Independent variable	*β*	*t*	*p*-value	*β*	*t*	*p*-value
CST state (preserved)	0.613	12.839	<0.001*	0.496	9.222	<0.001*
Diabetes (presence)	−0.21	−0.455	0.649	−0.13	−0.775	0.799
Age	−0.215	−4.518	<0.001*	−0.245	−4.564	<0.001*
Hemorrhage volume	−0.162	−3.065	0.002*	−0.190	−3.198	0.002*
Treatment (surgery)	0.154	2.855	0.005*	0.159	2.618	0.009*
*R* ^2^, *F* (*p*-value)	0.565, 55.869 (<0.001*)			0.449, 35.006 (<0.001*)		

Dependent variable	MBC			FAC		
Independent variable	*β*	*t*	*p*-value	*β*	*t*	*p*-value
CST state (preserved)	0.649	14.394	<0.001*	0.367	6.310	<0.001*
Diabetes (presence)	−0.008	−0.188	0.851	−0.011	−0.192	0.981
Age	−0.172	−3.816	<0.001*	−0.314	−5.411	<0.001*
Hemorrhage volume	−0.161	−3.222	0.001*	−0.237	−3.689	<0.001*
Treatment (surgery)	0.163	3.181	0.002*	0.126	1.915	0.057
*R* ^2^, *F* (*p*-value)	0.612, 67.861 (<0.001*)			0.345, 28.437 (<0.001*)		

Abbreviations: CST, corticospinal tract; MI, motricity indices; MBC, modified Brunnstrom classification; FAC, functional ambulation category.

^*^Statistically significant: *p* < 0.05.

In addition, there was no correlation between HbA1c level and motor outcome at 6 months after ICH onset (UMI: *r* = −0.096, *p* = 0.547; LMI: *r* = −0.169, *p* = 0.286; MBC: *r* = −0.088, *p* = 0.580; FAC: *r* = −0.190, *p* = 0.228).

## 4 Discussion

The current study evaluated the influence of diabetes on motor recovery in patients with ICH in the BG, adjusting for the CST integrity, age, hematoma volume, and treatment method.

Stroke type, lesion location and volume, and treatment method are important factors for motor prognosis in stroke patients [2,5,6,7]. The older the patient, the worse the prognosis [4]. Furthermore, CST integrity is a decisive factor in motor recovery [2,5,7]. Several DTT studies examining the CST in stroke patients showed that CST preservation is critical for motor recovery [2,7,17,18]. Therefore, using multiple linear regression analysis, we adjusted for these critical factors to accurately investigate the effects of diabetes on motor recovery after stroke. In our study, the CST state, age, lesion volume, and treatment method were significantly correlated with 6 month motor outcomes, a finding consistent with those of several previous studies. In 2002, Bagg et al. [19] prospectively evaluated the contribution of age to motor recovery in 640 patients, reporting that age was a significant predictor of functional independence at discharge after stroke treatment. Additionally, in 2008, Denti et al. [20] reported lower functional independent measurement scores upon discharge in stroke patients aged ≥75 years [21,22]. Moreover, the hematoma volume was significantly correlated with motor impairment after stroke. Large hematomas have a higher potential to damage brain areas that can contribute to motor recovery and motor-related neural tracts. Most ICHs do not require surgical treatment. However, early surgical removal of an intraparenchymal hematoma might be beneficial to reduce the mass effect, prevent or avoid midline shift, improve cerebral perfusion, and reduce the cascade of secondary brain injury [23]. Although the patients in our study who underwent surgery showed better motor outcomes than those who received conservative treatment, further studies are necessary.

In this study, diabetes did not affect 6 month motor outcomes, consistent with the results of some previous studies [8,9]. In 1995, Jørgensen et al. [8] evaluated the effect of diabetes on motor outcomes after stroke by comparing these outcomes at discharge following initial treatment for acute stroke in 176 and 902 patients with and without diabetes, respectively. They observed no significant differences in motor outcomes between these patient groups. In 2009, Nannetti et al. [9] measured the motor function of 93 stroke patients with diabetes and 302 stroke patients without diabetes after an average of 84.2 post-stroke days. They found that the scores (Barthel Index and Fugl–Meyer assessment scale) at the follow-up evaluations did not differ significantly between groups. In contrast to the results of our study, in 1983, Pulsinelli et al. [10] reported worse motor prognosis at discharge after cerebral infarct in 35 patients with diabetes compared with that in 72 patients without diabetes. Recently, in 2019, Moon et al. [7] analyzed the CST state using DTT in patients with corona radiata infarct. The patients were grouped based on the CST state and the presence of diabetes. They found that when the CST was interrupted, the presence of diabetes was a decisive factor in the patient’s motor recovery. However, the authors did not consider the influence of lesion volume or age. Lesion volume is a critical factor that can affect motor outcome after stroke; thus, their study is limited as they did not consider factors that should have been adjusted for. Other than Moon’s study, the other studies mentioned above did not consider the CST state, lesion location, age, lesion volume, or treatment method.

Although diabetes may inhibit motor recovery mechanisms such as perilesional reorganization and contributions from the secondary motor area, our results showed that diabetes did not significantly hinder motor recovery in stroke patients.

In conclusion, we found that diabetes did not significantly affect motor outcomes after ICH. Our study is the first to evaluate the effect of diabetes on motor recovery after stroke that controlled for multiple critical factors including the state of motor-related neural tracts, lesion location, age, lesion volume, and treatment method. However, our study is limited in that it was conducted retrospectively. Well-controlled prospective studies are warranted to clarify the effect of diabetes on motor recovery after stroke.
